# Duplex collecting system with ectopic ureter in adult: a case report and literature review

**DOI:** 10.1097/MS9.0000000000003726

**Published:** 2025-08-14

**Authors:** Lei Yang, Rui Jiang, Yuxuan Tian, Yang Yang, Wei Yu

**Affiliations:** a Department of Urology, Peking University First Hospital, Peking University, Beijing, China; b Department of Urology, Institute of Urology, Peking University, Beijing, China; c Department of Urology, National Urological Cancer Center, Beijing, China

**Keywords:** duplicated kidney, ectopic ureter, urinary incontinence

## Abstract

**Introduction and importance:**

Bilateral renal collecting system duplication is a rare condition in urology, and even rarer is the presence of an ectopic ureter. We described a case of bilateral renal collecting system duplication with an ectopic left-sided ureter opening into the urethra. The left side displayed complete renal collecting system duplication, while the right side showed incomplete duplication.

**Case presentation:**

55-year-old female was diagnosed with bilateral renal collecting system duplication and an ectopic left-sided ureter opening into the urethra. She underwent cystourethroscopy and ureteral-bladder anastomosis at our hospital. The patient was discharged after 5 days post-operation without significant discomfort. One month later, the double-J catheter was removed, and she reported no further incontinence during follow-up.

**Clinical discussion:**

Urinary incontinence is the most common complaint in females with ectopic ureters, as the ureteral opening bypasses the external urethral sphincter. Complete renal collecting system duplication is often associated with an ectopic ureter, resulting in persistent urinary incontinence. Notably, the patient did not experience incontinence during childhood, with intermittent incontinence only appearing over the past 3 years. For this patient, exploration surgery clarified the anatomical abnormality, and imaging showed that the upper pole of the left kidney had an acceptable cortical thickness, thus the decision was made to perform ectopic ureteral bladder reimplantation, which avoided excessive resection of the duplicated kidney and unnecessary anti-stress incontinence surgery. Therefore, for complicated cases, a staged treatment strategy is recommended, starting with exploratory surgery to clarify the patient’s specific anatomy before determining the subsequent surgical approach.

**Conclusion:**

Bilateral renal collecting system duplication with an ectopic left-sided ureter opening into the urethra is extremely rare. Ureteral reimplantation can effectively reconstruct the urinary tract, and sometimes multiple surgeries are required, which should be chosen according to the patient’s specific situation.

## Introduction

**Figure 1.**
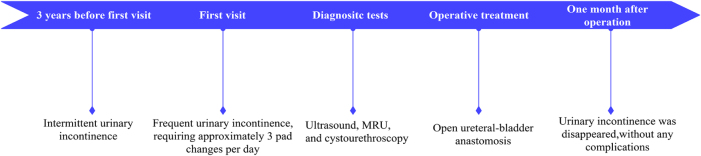
The timeline of clinical courses of the patient.

Renal collecting system duplication is a relatively common congenital anomaly where one kidney has two separate collecting systems and ureters, typically occurring unilaterally, with bilateral cases being rare; bilateral renal collecting system duplication combined with an ectopic ureter opening into the urethra is extremely rare^[1]^. An ectopic ureter is a condition where the ureter drains into an abnormal location, often leading to urinary incontinence or infection. Ureteral reimplantation is a surgical procedure where the ectopic ureter is surgically repositioned to the bladder to restore normal urinary flow^[2]^. We reported a case of a female patient with bilateral renal collecting system duplication and an ectopic left-sided ureter opening into the urethra. The patient has congenital bilateral renal collecting system duplication, with the left side fully duplicated and the right side incompletely duplicated. We reviewed the existing literature and shared our diagnostic and treatment experience with this case. The timeline of clinical courses of the patient is illustrated in Fig. 1.

## Case presentation

The patient is a 55-year-old female admitted with a history of urinary incontinence lasting over 3 years. Three years ago, she presented with involuntary leakage of urine when abdominal pressure increased. She had no history of urinary leakage during her youth but had undergone cystoscopy at another hospital, which revealed a urinary tract anomaly before considering surgery for stress urinary incontinence. She declined surgery and did not receive any other treatment. Over the past year, her incontinence worsened, requiring approximately 3 pad changes per day, significantly affecting her daily life. She had no issues with normal urination. Upon examination, it was noted that urinary spurt was observed on Valsalva maneuver, even in the absence of a full bladder, and her pelvic muscle strength was normal. No obvious urine leakage was noted when she coughed while performing a pelvic floor contraction. Her external urethral opening and vaginal morphology were normal. She was treated at our hospital.

**Figure 2.**
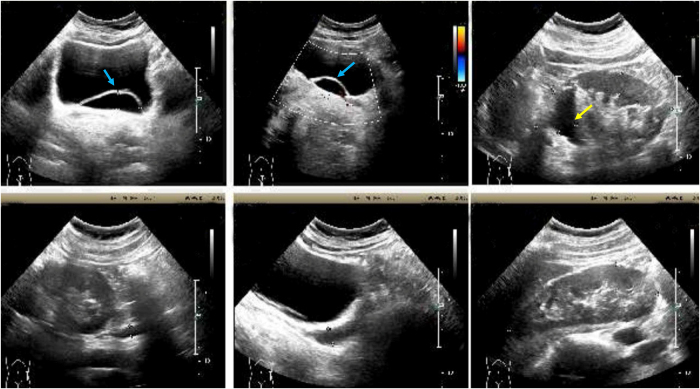
Left duplicated kidney, with upper kidney and ureter showing dilated hydronephrosis (blue arrow); left ureteral orifice ureterocele (yellow arrow).

**Figure 3.**
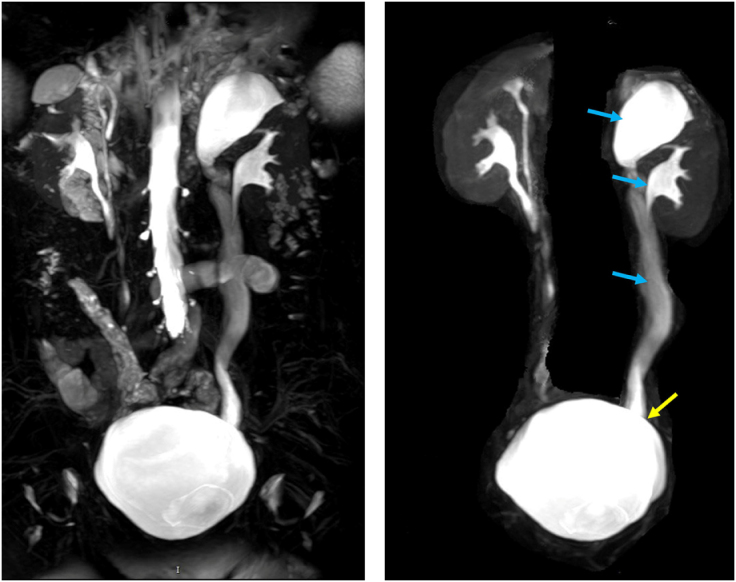
Bilateral renal pelvis and ureteral anomalies. Left upper renal pelvis, calyces, and ureter showing dilatation (blue arrow), with the left ureteral distal end appearing to drain into the posterior lower wall of the bladder (yellow arrow).

Ultrasound (Fig. 2) showed two sets of collecting systems on the left side, with a thickness of 0.39 cm in the upper kidney parenchyma and severe hydronephrosis, with the upper ureter dilated along its entire length. A cystic anechoic structure was observed at the left ureteral orifice on the posterior wall of the bladder, measuring 5.45 × 1.48 cm, with thin walls. Enhanced MRI and MRU (Fig. 3) revealed bilateral renal pelvicalyceal duplication, with thinning of the upper pole of the left kidney. The left upper renal pelvis, calyces, and ureter were dilated, with no contrast entering during the secretion phase, and the distal part of the left ureter appeared to drain into the left posterior lower wall of the bladder.

**Figure 4.**
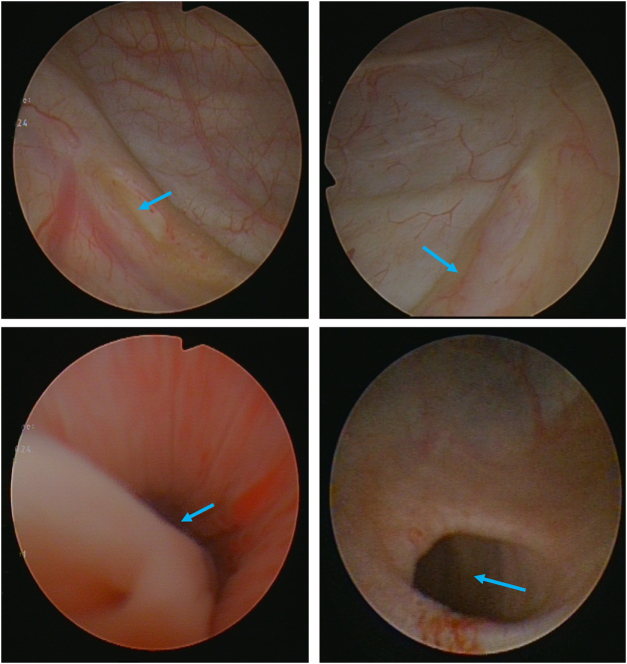
(a) Normal right ureteral orifice position. (b) Normal left ureteral orifice position. (c) Ectopic opening in the urethra. (d) Ureterocele and ureter within the ureterocele.

The patient had no other systemic diseases, denied a family history of similar diseases or congenital conditions, and routine blood, urine, biochemical, and coagulation tests showed no significant abnormalities. No urinary tract infection or renal dysfunction was noted. Based on these tests, the initial diagnosis was stress urinary incontinence, possibly due to either the usual female stress urinary incontinence or a rare case of an ectopic ureter opening at the distal external urethral sphincter, or a combination of both. Imaging studies indicated bilateral renal collecting system duplication, with complete duplication on the left side and incomplete duplication on the right side. The position of the left ectopic ureteral opening was unclear. Thus, the patient underwent cystourethroscopy and ureteral-bladder anastomosis.Cystourethroscopy: During the procedure, the right and left ureteral orifices were found at their normal positions (Fig. 4a, 4b). A thin-walled cystic structure was seen at the left bladder trigone, consistent with a ureterocele, but no orifice was detected within the bladder. Upon approaching the external urethral opening, the ectopic ureteral orifice was located (Fig. 4c), leading into a widened ureterocele (Fig. 4d), which is distal end of the left lower pole ureter. This confirmed the presence of an ectopic ureter opening at the distal sphincter. However, it was uncertain whether the urinary incontinence was related to an anatomical abnormality of the urethra. Following communication with the patient, the decision was made to first address the ectopic ureteral opening, with further evaluation of stress urinary incontinence to decide whether additional surgery was necessary.

**Figure 5.**
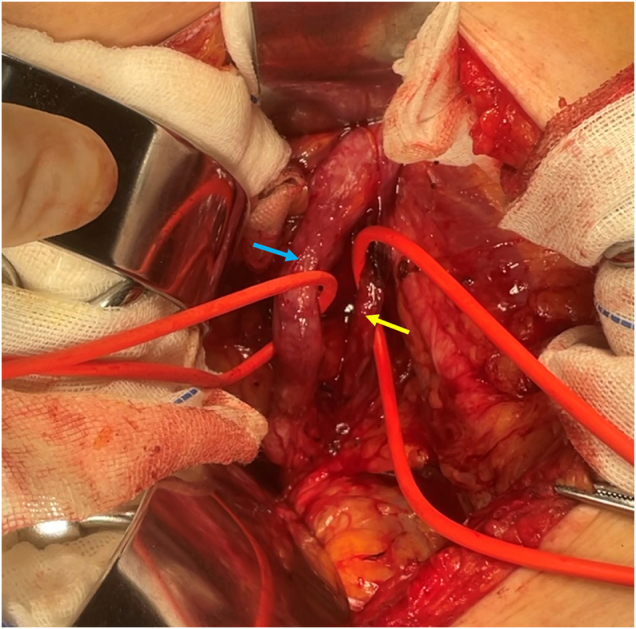
Left-sided ectopic ureter (blue arrow) and normal ureter (yellow arrow) intertwined and descending together.

Ureteral-bladder anastomosis: A protective F5 ureteral stent was placed at the left normal ureteral orifice during cystourethroscopy, and the procedure was carried out smoothly. Considering her history of caesarean section and subsequent uterine polypectomy, the open surgical approach is selected for better visibility and tactile feedback. The McBurney incision provides excellent access to the right lower quadrant, where the ectopic ureter and associated anomalies are located. This allows for precise dissection of the anatomical structures involved in the duplex collecting system and ectopic ureter without excessive manipulation of surrounding tissues. Thus, a McBurney incision was made, and the retroperitoneal space was accessed. The dilated ectopic left-sided ureter, which had entwined with the normal ureter, was carefully separated (Fig. 5). The ectopic ureter was freed up to the level near the bladder top, where the distal end was ligated and the proximal end was anastomosed to the bladder. A 6-26 DJ catheter was selected for this patient with a medium to tall stature (165 cm), as it provided the necessary length and flexibility for proper positioning within the renal collecting system and ensures adequate drainage of urine from the kidney into the bladder. A catheter was inserted into the proximal end of ectopic ureter, which was sutured and reimplanted at the bladder apex at the determined reimplantation site. The total operative time for the procedure was approximately 110 minutes. The estimated blood loss was minimal, approximately 10 mL. The patient was discharged on postoperative day 5, without any complications, such as infection, bleeding, or urinary tract injury. According to the Clavien–Dindo classification, the patient’s outcome would be classified as Grade 0, which denotes no complications.

**HIGHLIGHTS**

- An explanation proposed for patients with ectopic ureters, who did not experience persistent urinary incontinence in childhood but developed intermittent urinary incontinence, is that the small ectopic ureter opening, surrounded by urethral muscle, initially formed a functional sphincter and prevented incontinence; however, with age and hormonal changes, the related muscles atrophied, leading to relaxation of the ectopic ureter opening and subsequent incontinence.
- For patients with complex conditions, a staged treatment strategy is recommended. Initial exploratory surgery should be performed to clarify the patient’s specific anatomy before developing the subsequent surgical plan. For this patient, unnecessary anti-stress incontinence surgery was avoided.
- Ultrasound could not detect the incomplete duplication of renal collecting system and fails to define the opening of the ectopic ureter. Through MRU and 3D reconstruction, the anatomical abnormalities of bilateral renal collecting system duplication were clearly demonstrated.
- Due to the variability in anatomical abnormalities within renal collecting system duplication, individualized approaches are necessary, and the surgical plan should be tailored based on the location of the obstruction and associated complications.

**Figure 6.**
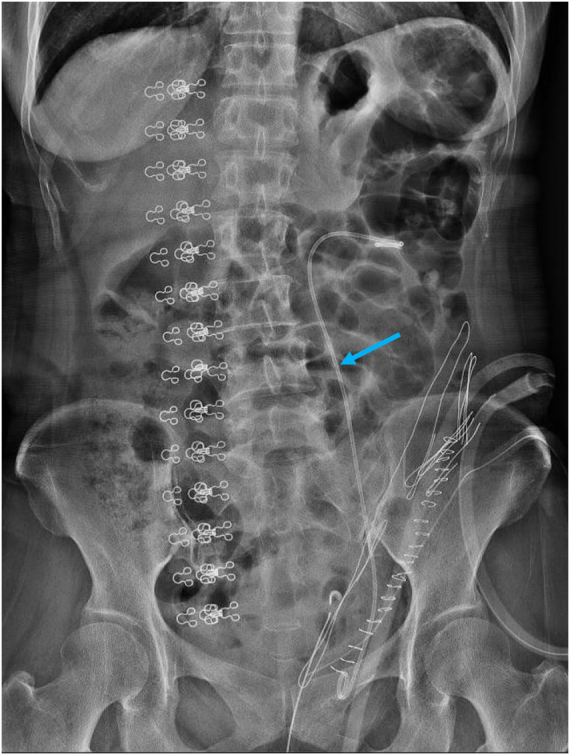
Normal placement of left double-J stent in post-operation.

The patient was monitored in the immediate postoperative period for any signs of complications, such as urinary retention, infection, or renal function decline. Post-operative KUB X-ray (Fig. 6) showed good positioning of the double-J stents. The patient received routine treatments, including fluid replacement, dressing changes, and anti-infection measures. One month after discharge, she underwent a follow-up ultrasound at a local hospital to evaluate the hydronephrosis. The results of the urinary tract ultrasound indicated significant improvement in the hydronephrosis, with the findings showing relief of the previously observed renal dilation. The left double-J catheter was removed without discomfort, and the patient reported that her urinary incontinence had disappeared. This confirmed that the stress urinary incontinence was caused by the ectopic ureter opening.

## Discussion

The work has been reported in line with the SCARE 2025 criteria^[3]^.

We reported a case of bilateral renal collecting system duplication with an ectopic left-sided ureter opening near the external urethral opening, where the patient presented with urinary incontinence that required surgical treatment to alleviate symptoms. We also conducted a literature review and discussed cases of adult renal collecting system duplication associated with ectopic ureters,^[4–19]^ providing an overview of the clinical phenotypes, anatomical differences, and treatment strategies for this patient group (Table 1).

**Table 1:** Literature review of adult patients with duplex collecting system and ectopic ureter

Age sex	Presentation	Anatomic anomalies	Management	Complications	Ref
54 male	Right flank pain after a fall	Duplicate left collecting system with left upper moiety blind-ended near prostatic fossa	Cystoscopy. Exploratory laparotomy	Not reported	^[4]^
35 male	Right upper quadrant pain	Duplicate right collecting system with right ectopic ureterocele extending into the prostatic fossa	Surgery	Not reported	^[5]^
40 female	Vague abdominal discomfort	Duplex right collecting system with right upper moiety inserted ectopically into the vagina	Right upper-pole hemi-nephrectomy	Not reported	^[6]^
47 female	Acute left flank pain and urinary tract infections.	Duplex left collecting system with left upper moiety inserted ectopically into the vagina	Conservative management	Not reported	^[6]^
43 female	Left flank pain	Duplicate bilateral collecting systems with left ectopic ureters opening to the bladder neck	Surgical resection of left lower collecting system including the kidney and ureter	Not reported	^[7]^
24 male	Right renal colic	Duplicate right collecting system with right ectopic upper inserted into the seminal vesicle	Stenosed infundibulum of right upper pole dilated with balloon	Repeat hospitalizations for sepsis	^[8]^
18 male	Recurrent left loin pain	Duplex left kidney with ectopic insertion of left upper ureter into verumontanum	Dilation of ectopic ureteral orifice	Asymptomatic at 5 years	^[9]^
27 male	Abdominal pain, distension, and fever	Duplex left kidney with ectopic ureter insertion into prostate	Left partial nephrectomy, and ectopic ureterectomy	None	^[10]^
72 male	Sepsis	Duplex left collecting system with ectopic upper-pole ureter insertion into prostatic urethra	Percutaneous nephrostomy tube insertion	Repeat hospitalizations followed. Patient died 9 months later	^[11]^
19 female	Right lower quadrant pain and dysuria	Duplex right ureter with ectopic ureter insertion into bladder base	Oral antibiotics and conservative urologic management	Lost to follow-up	^[12]^
25 female	Mild recurrent right flank pain	Duplex right ureter with upper pole ureteral insertion in posterior urethra	Cystoscopy, re-admitted for laparoscopic hemi-nephrectomy	No complications following hemi-nephrectomy	^[13]^
20 female	Fever, fatigue, nausea, anorexia, epigastric pain, right flank pain	Duplex right collecting system with right upper-pole moiety insertion between the urethra and anterior vagina	Antibiotics	Not reported	^[14]^
17 female	Continuous dribbling of urine since childhood	Duplex bilateral collecting systems with right ectopic ureter draining below bladder neck into upper urethra	Right upper pole hemi nephroureterectomy	No symptoms	^[15]^
24 female	Constant loss of urine since childhood	Duplicate left ureteral with an ectopic ureter opening into the vulva	Cystoscopy, upper pole partial nephrectomy,	No episodes of urinary incontinence	^[16]^
43 female	Acute left flank pain associated with a burning sensation during micturition	Duplex left collecting system with left upper moiety inserted ectopically into the vagina	Cystoscopy, surgical re-implantation of the left upper moiety	Not reported	^[17]^
60 male	Intermittent fever, right flank soreness, dysuria, and hematuria	Duplex right collecting system with right upper moiety located inferomedially to the orthotopic ureter	Percutaneous nephrostomy, transurethral resection of the ureterocele	Resolution of hydronephrosis without newly developed vesicoureteral reflux	^[18]^
28 female	Lifelong urinary leakage since birth	Duplex left collecting system with left upper moiety implanted ectopically into the vagina	Surgical re-implantation of both left ureters into the bladder	Marked improvement in urinary symptoms	^[19]^

While renal collecting system duplication is a relatively common congenital condition, bilateral renal collecting system duplication with an ectopic ureter opening into the urethra is exceedingly rare. The specific incidence of ectopic ureters opening into the urethra is not well-defined in the literature, generally representing only 10–20% of all cases of ectopic ureters, with the condition being significantly more common in females than in males, typically diagnosed in childhood or adolescence^[1]^. In incomplete renal collecting system duplication, two sets of collecting systems and two ureters merge at any location between the kidney and bladder, forming a single normal ureter that inserts into the bladder. In complete renal collecting system duplication, there are two fully independent ureters and renal pelvises^[20]^. According to the Weigert–Meyer rule, the upper part of the duplicated ureter opens medially and inferiorly, while the lower part opens laterally and superiorly^[21]^. In females, the most common sites of ectopic ureter openings are the bladder neck, urethra, vestibule, vagina, and uterus^[22]^. In this case, the left renal collecting system exhibited complete duplication, with the upper ureter opening near the external urethral opening and the lower ureter opening at the normal bladder position, in accordance with the Weigert–Meyer rule.

Renal collecting system duplication can be asymptomatic, but complications such as abdominal pain, hematuria, urinary stones, urinary tract infections, vesicoureteral reflux, urinary incontinence, ureteroceles, and ectopic ureters may arise. In cases of complete renal collecting system duplication, the upper ureter often opens ectopically into the vagina, urethra, epididymis, or vestibule, leading to persistent urinary incontinence and continuous dribbling, and may resulting in ureteroceles^[23]^. In incomplete renal collecting system duplication, vesicoureteral reflux, ureter-ureter reflux, and obstruction at the renal-pelvic junction are common^[24]^. Although the right side of this patient presented as incomplete duplication, no reflux or obstruction was observed on the right side; the left side, however, exhibited complete duplication with an ectopic ureter opening into the urethra, presenting with urinary incontinence. Urinary incontinence is the most common complaint in patients with ectopic ureters, particularly in females, as the ectopic opening bypasses the external sphincter. Male patients may not experience incontinence because the ectopic opening is usually above the external sphincter^[25]^. It is crucial to determine whether the incontinence is continuous or intermittent for accurate diagnosis, as persistent urinary incontinence often indicates urethral anatomical anomalies such as ectopic ureters, most of which are associated with complete renal collecting system duplication^[26]^. Interestingly, this patient did not experience urinary incontinence in childhood, and the intermittent urinary incontinence only developed in the past three years. The potential explanation of the mechanism behind delayed-onset incontinence is that the small ectopic ureter opening, surrounded by urethral muscle, initially formed a functional sphincter, preventing incontinence; however, with age and hormonal changes, sphincter is atrophied and incompetent, leading to relaxation of the ectopic ureter opening and subsequent incontinence^[27,28]^. Besides, a differential diagnosis for mixed stress urinary incontinence is necessary to aid in more accurate clinical evaluation. According to history-taking, symptom assessment, and physical examination, this patient often describe leakage after increased intra-abdominal pressure, without a sudden, overwhelming need to urinate, since urge urinary incontinence could be excluded. Moreover, when she was performing a pelvic floor contraction, coughing fails to elicit obvious urine leakage, suggesting that it is not pure stress urinary incontinence. Thus, further imagological examinations and cystourethroscopy are performed to check possible abnormalities of the urinary tract structure.

The diagnosis of renal collecting system duplication relies heavily on imaging examinations. Ultrasound is the preferred method, as it can show uneven renal hydronephrosis between the upper and lower poles of the duplicated kidneys, as well as ureteroceles at the ectopic ureter’s distal end^[29]^. However, ultrasound cannot detect the location of the ectopic ureter opening and does not provide a clear description of the anatomical relationship between the ureter, bladder, urethra, and vagina^[30]^. In addition, ultrasonography fails to reveal the full extent of the anatomic abnormalities. The limitations of ultrasonography in visualizing complex anatomical structures, such as a duplex collecting system with an ectopic ureter, can lead to diagnostic ambiguity, especially when the pathology is subtle or when the collecting system has a non-standard configuration. Therefore, CT or MR urography should be used to clarify the location and course of the ectopic ureter^[31]^. MRU provides superior soft-tissue contrast and allows for the precise identification of the duplex collecting system, the ectopic insertion of the ureter, and any associated anomalies. MRU also allows for visualization of the relationship between the renal parenchyma and the collecting system, which is difficult to assess using traditional imaging modalities like ultrasonography or intravenous pyelography. In this patient’s diagnostic process, ultrasound revealed severe hydronephrosis in the left duplicated kidney but failed to detect the incomplete duplication on the right side. It also identified a ureterocele at the distal end of the ectopic ureter, but the opening could not be clearly defined. Through MRU and 3D reconstruction, the anatomical abnormalities of bilateral renal collecting system duplication were clearly demonstrated. Urodynamic studies are essential for evaluating bladder function, including detrusor pressure, bladder capacity, and sphincter function. In this case, urodynamics could have been used to confirm whether the incontinence was truly due to a lack of sphincter integrity or due to an anatomic abnormality, such as the ectopic ureter, that might lead to involuntary leakage during physical exertion. In this case, patient refused this invasive examination, but urodynamic testing would have been a valuable step to definitively rule out pure stress incontinence. Inaccurate imaging and the failure to properly differentiate between true stress urinary incontinence and incontinence caused by anatomical abnormalities, such as a duplex collecting system or ectopic ureter, can lead to inappropriate treatment decisions, including unnecessary surgical interventions like sling procedures or periurethral injections. These procedures carry risks of complications, including urinary retention, infection, and voiding dysfunction. Furthermore, the patient’s underlying urological conditions may remain untreated, leading to persistent symptoms and potential deterioration of renal function. The importance of advanced imaging and functional studies is crucial to avoid these pitfalls and ensure that the patient receives the most appropriate therapy. Inaccurate imaging and the failure to properly differentiate between true stress urinary incontinence and incontinence caused by anatomical abnormalities, such as a duplex collecting system or ectopic ureter, can lead to inappropriate treatment decisions, including unnecessary surgical interventions like sling procedures or periurethral injections. These procedures carry risks of complications, including urinary retention, infection, and voiding dysfunction. Furthermore, the patient’s underlying urological conditions may remain untreated, leading to persistent symptoms and potential deterioration of renal function. The importance of advanced imaging \and functional studies is crucial to avoid these pitfalls and ensure that the patient receives the most appropriate therapy. For patients with complex conditions, a staged treatment strategy is recommended. Initial exploratory surgery should be performed to clarify the patient’s specific anatomy before developing the subsequent surgical plan. For this patient, unnecessary anti-stress incontinence surgery was avoided.

Most patients with renal collecting system duplication are asymptomatic and therefore do not require treatment. However, for symptomatic patients, treatment approaches vary. For those with symptomatic cases and non-functional duplicated kidneys or ectopic ureters, most urologists perform laparoscopic partial nephrectomy. If the duplicated kidney is functional, surgical options such as pyeloureterostomy, ureteroureterostomy, and ureteral-bladder reimplantation are available to preserve renal parenchyma. These procedures can be performed using either laparoscopic or open surgical techniques^[32–34]^. For patients with ectopic ureteroceles who have urinary tract obstruction, the placement of a double-J stent through endoscopic techniques may be an effective method for relieving the obstruction^[35]^. In this case, the left duplicated kidney still had an acceptable cortical thickness, indicating that renal function is preserved to some degree, so ureteral-bladder reimplantation was chosen. Minimally invasive option, such as laparoscopic ureteroneocystostomy, could become an alternative that offer benefits in terms of recovery time and patient outcomes^[36,37]^. Postoperatively, the patient’s urinary incontinence symptoms resolved with no significant complications. Therefore, due to the variability in anatomical abnormalities within renal collecting system duplication, individualized approaches are necessary, and the surgical plan should be tailored based on the location of the obstruction and associated complications.

## Conclusion

Bilateral renal collecting system duplication with an ectopic ureter is a rare congenital anomaly, primarily seen in females. Ultrasound is the preferred method for detecting renal collecting system duplication, while CT or MR urography can delineate the anatomical relationships of the urinary and genital organs and determine the location of the ectopic ureter. In patients with functionally preserved duplex kidneys, ureteral reimplantation may be chosen as a reconstructive procedure to restore normal urinary tract anatomy, effectively alleviating urinary incontinence.

## Data Availability

No other datasets were generated during and/or analyzed during the current study. All the information is available with the manuscript.
